# Speckle-tracking carotid strain ultrasonography for assessment of vascular dysfunction in metabolic syndrome: a cross-sectional study

**DOI:** 10.1186/s12880-026-02686-5

**Published:** 2026-09-11

**Authors:** Elif Duygu Topan, Aral Varol, Hilal Bektaş Uysal, İmran Kurt Ömürlü, Mustafa Gök

**Affiliations:** 1 Department of Internal Medicine, Aydın Adnan Menderes University Faculty of Medicine, Aydın, Türkiye; 2 Department of Radiology, Aydın Adnan Menderes University Faculty of Medicine, Aydın, Türkiye; 3 Department of Biostatistics, Aydın Adnan Menderes University Faculty of Medicine, Aydın, Türkiye

**Keywords:** Arterial stiffness, Metabolic syndrome, Speckle-tracking carotid strain, Carotid ultrasonography, Subclinical atherosclerosis

## Abstract

**Background:**

Metabolic syndrome (MetS) is associated with increased cardiovascular risk, which rises with the number of MetS components. Conventional carotid ultrasound markers may not fully characterize dynamic arterial wall mechanics, whereas speckle-tracking carotid strain ultrasonography (STCS-US) enables integrated assessment of carotid stiffness and deformation throughout the cardiac cycle. This study aimed to assess multidimensional carotid mechanics in individuals with MetS and their association with cumulative metabolic burden.

**Methods:**

This retrospective cross-sectional study included 100 participants:50 patients with MetS diagnosed according to the National Cholesterol Education ProgramAdult Treatment Panel III (ATP III) criteria and 50 age and sex matched controls without MetS Carotid Doppler US images were analyzed using the Arterial Analysis software program, which quantified 22 vascular parameters per participant, comprising five stiffness parameters (β-stiffness index [β-SI], arterial compliance [AC], arterial distensibility [AD], elastic modulus [EM], and pulse wave velocity [PWV]) and six strain parameters (radial strain, radial displacement [DP], radial strain rate [SR], circumferential strain, circumferential displacement, and circumferential strain rate), each obtained in both the longitudinal and transverse planes. Correlations between the number of MetS components and imaging-derived vascular parameters were analyzed. Classification and Regression Trees (CART) analysis was performed to evaluate the relative contribution of clinical and structural variables to STCS-US-derived vascular parameters.

**Results:**

Patients with MetS exhibited significantly increased stiffness parameters, including EM and PWV, and reduced compliance indices compared with controls (all *p* < 0.05). Strain analysis demonstrated significantly lower radial and circumferential strain percentages in the MetS group, with more pronounced alterations observed in the transverse plane (all *p* < 0.001). Increasing MetS component count was associated with higher stiffness indices and lower strain values. CART analysis demonstrated that MetS status remained one of the most important determinants across the reported STCS-US-derived vascular parameters.

**Conclusion:**

STCS-US revealed coordinated impairment in carotid arterial stiffness and strain parameters in MetS, particularly in the transverse plane. These findings suggest that STCS-US may provide complementary functional information alongside conventional stiffness assessment and may reflect the cumulative metabolic burden on vascular mechanics.

**Supplementary Information:**

The online version contains supplementary material available at https://doi.org/10.1186/s12880-026-02686-5.

## Background

Metabolic syndrome (MetS) is characterized by the clustering of central obesity, insulin resistance, dyslipidemia, and hypertension, and affects approximately one quarter of the adult population worldwide [1]. This highly prevalent condition is strongly associated with an increased risk of cardiovascular disease and all-cause mortality, with cardiovascular risk rising in parallel with the number of MetS components [2]. The cumulative burden of these metabolic abnormalities contributes substantially to global cardiovascular morbidity, underscoring the need for early identification of vascular dysfunction before overt clinical disease develops [3].

Arterial stiffness is considered a key mechanism linking MetS to adverse cardiovascular outcomes. Chronic low-grade inflammation, oxidative stress, and endothelial dysfunction contribute to vascular remodeling and reduced arterial elasticity in individuals with MetS [4]. Pulse wave velocity (PWV), a widely accepted measure of arterial stiffness, is an established independent predictor of cardiovascular events [5, 6]. Conventionally, arterial stiffness is assessed using applanation tonometry, which is considered the gold standard technique for the assessment of arterial stiffness [7]. However, conventional structural markers such as carotid intima-media thickness (CIMT) and plaque burden primarily reflect morphological changes and may not fully capture early functional alterations in arterial wall mechanics.

Functional vascular parameters of the common carotid artery, including elastic modulus, distensibility, and strain-based measures, may detect early impairment before overt structural remodeling becomes apparent [7]. These non-invasive indices have demonstrated prognostic relevance in selected populations, but their integrated assessment in patients with MetS remains incompletely characterized.

Speckle-tracking carotid strain ultrasonography (STCS-US) enables quantification of carotid arterial dysfunction throughout the cardiac cycle and provides functional assessment of arterial mechanics beyond conventional stiffness indices. Prior studies have demonstrated the feasibility of strain-based carotid assessment in the general population and have suggested associations between reduced carotid strain and adverse cardiovascular outcomes [8, 9]. However, data evaluating the integrated assessment of carotid stiffness and strain parameters specifically in individuals with MetS remain limited.

Therefore, we aimed to examine the association between MetS and carotid stiffness and strain parameters measured using STCS-US.

## Methods

### Ethical approval

This study was conducted in accordance with the ethical principles of the Declaration of Helsinki (1964) and its later amendments. The study protocol was approved by the Non-Interventional Clinical Research Ethics Committee of Aydın Adnan Menderes University (approval date: 27.06.2025; protocol no: 2025/220). Owing to the retrospective design, the requirement for informed consent was waived by the ethics committee. All patient data were anonymized, and confidentiality was strictly maintained throughout the study.

### Study population and sample selection

A total of 100 participants (57 females, 43 males) were included in the study, comprising 50 patients with MetS and 50 controls without MetS. Based on an a priori power analysis referencing Lopes-Vicente et al. [10], with an alpha of 0.01, an effect size of 0.8, and a statistical power of 90%, a minimum of 37 subjects per group was determined to be sufficient, indicating that the final sample size was adequate. Patients who met the diagnostic criteria for MetS and had archived carotid Doppler US cine-loop images available in the institutional Picture Archiving and Communication System (PACS) (Sectra Workstation IDS-7, Version: 26.2.12.8350, Sectra AB Teknikringen 20 SE-583 30 Linköping, SWEDEN) between January 2025 and June 2025 were retrospectively identified and included in the study. The control group consisted of age and sex matched individuals without MetS who also had archived carotid Doppler US examinations available for analysis.

### Inclusion and exclusion criteria

**Fig. 1:**
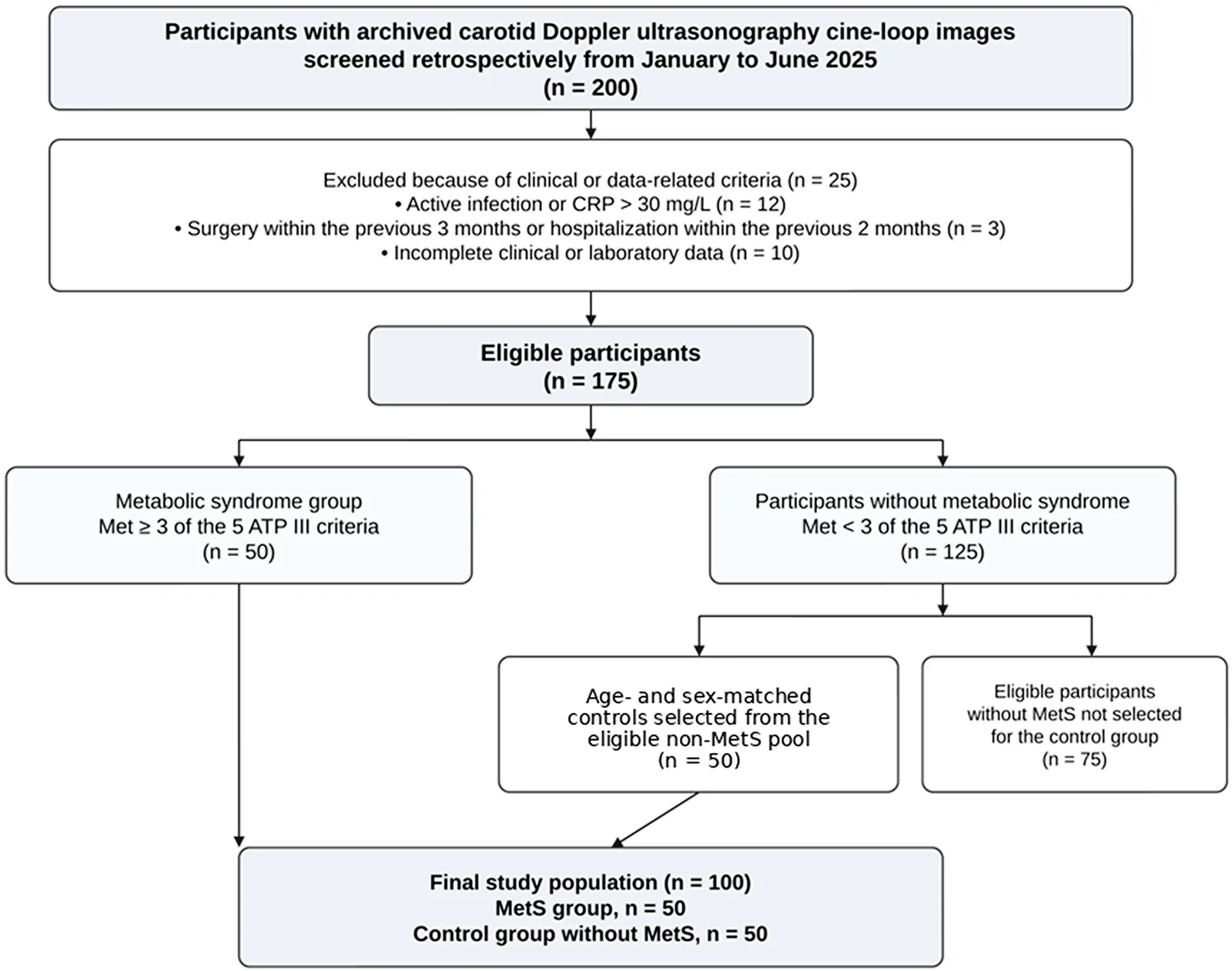
Flowchart of the study population. MetS: metabolic syndrome; CRP: C−reactive protein; ATP III: adult treatment panel III

Individuals were diagnosed with MetS according to the operational criteria of the National Cholesterol Education Program, Adult Treatment Panel III (ATP III) [11], which define MetS as the presence of at least three of the following five risk factors: waist circumference (WC) ≥ 102 cm for men or ≥ 88 cm for women, triglycerides (TG) ≥ 150 mg/dL, high-density lipoprotein cholesterol (HDL-C) < 40 mg/dL for men or < 50 mg/dL for women, systolic blood pressure (SBP) ≥ 130 mmHg or diastolic blood pressure (DBP) ≥ 85 mmHg, and fasting blood glucose (FBG) ≥ 100 mg/dL, consistent with impaired fasting glucose or type 2 DM. Patients aged 18–80 years who met at least three of the five ATP III criteria were included in the MetS group, whereas the control group consisted of age- and sex-matched individuals who did not meet the ATP III definition of MetS. Exclusion criteria were the presence of active infection, C-reactive protein (CRP) level above 30 mg/L, a history of surgical intervention within the past three months, or hospitalization within the past two months. The inclusion and exclusion process of the study population is summarized in Fig. 1.

### Data collection

A structured case report form was developed by the investigators based on a review of relevant literature. The form included demographic information (age, sex, smoking status), anthropometric measurements (height, weight, body mass index (BMI), WC), and vital signs (SBP, DBP). Laboratory parameters were obtained from the hospital’s electronic medical record system (MIA-MED Hospital Information System, Version 1.0.1.5000, MIA Teknoloji, Ankara, Türkiye) and included FBG, lipid profile (total cholesterol, low-density lipoprotein cholesterol (LDL-C), HDL-C, TG), renal function tests (blood urea nitrogen, creatinine, estimated glomerular filtration rate), liver enzymes (aspartate aminotransferase (AST), alanine aminotransferase (ALT)), uric acid, and hematologic indices (hemoglobin, white blood cell count (Wbc), neutrophil count (Neu), lymphocyte count (Lym), and platelet (Plt) count).

Archived carotid Doppler US cine-loop images were retrospectively retrieved from the institutional PACS and analyzed using the Arterial Analysis software program (Samsung Medison Co., Ltd., Seoul, Korea). Carotid US examinations had originally been performed using a Samsung V7 US system equipped with an LA2-14 A linear probe (Samsung Medison Co., Ltd., Seoul, Korea).

The Arterial Analysis software was used to obtain arterial stiffness and strain parameters. Stiffness parameters included β-stiffness index (β-SI), arterial compliance (AC), arterial distensibility (AD), elastic modulus (EM), and pulse wave velocity (PWV), which were obtained in both longitudinal and transverse planes. Strain parameters included radial displacement (DP), radial strain (%), and radial strain rate (SR) obtained in both longitudinal and transverse planes, as well as circumferential displacement (DP), circumferential strain (%), and circumferential strain rate (SR) obtained in the transverse plane.

Although routine carotid Doppler US included evaluation of the entire carotid system, STCS-US analyses in the present study were performed using archived cine-loop images obtained from the distal common carotid artery segment immediately below the carotid bulb. This predefined segment was used for all measurements in all participants. Control points were manually placed, and arterial wall displacement was tracked using the integrated optical flow algorithm. Segments containing plaques, calcifications, acoustic shadowing, or inadequate tracking quality were excluded from analysis. The mean of the right and left carotid measurements was used for statistical analysis.

### Statistical analysis

Data were analyzed using SPSS version 26.0 (IBM Corp., Armonk, NY, USA). The distribution of continuous variables was assessed using the Kolmogorov–Smirnov test and visual inspection of histograms and Q-Q plots. Normally distributed variables were compared with the independent sample t test, while non-normally distributed variables were compared with the Mann–Whitney U test. Categorical variables were analyzed using the Chi-square test. Descriptive statistics are presented as mean±standard deviation, median (25th–75th percentiles) or frequency (%). The correlations between the number of MetS diagnostic criteria and vascular parameters were assessed using Spearman’s correlation test.

To evaluate the relative contribution of clinical and structural variables to STCS-US-derived vascular parameters, Classification and Regression Tree (CART) analysis was performed. MetS status, age, sex, BMI, smoking status, CIMT, plaque presence, antidiabetic treatment, antihypertensive treatment, and antihyperlipidemic treatment were included as candidate determinants. To ensure model generalizability and mitigate the risk of overfitting, a 3-fold cross-validation scheme was applied during the tree-building process. Model performance and tree optimization were evaluated using mean absolute error (MAE) and mean absolute percentage error (MAPE). Variable importance scores were calculated for each model and normalized to a maximum value of 100%. The final optimized model structures were visualized using decision-tree diagrams to illustrate the hierarchical splitting process.

To assess measurement reliability, an intra-observer reproducibility analysis was performed. The intraclass correlation coefficient (ICC) and coefficient of variation (CV) were calculated.

## Results

Baseline demographic and laboratory characteristics of the study population are summarized in Table 1. The study included 100 participants, comprising 50 patients with MetS and 50 age and sex matched healthy controls There were no significant differences between the two groups in terms of age, sex distribution, hemoglobin, platelet count, creatinine, or smoking status (all *p* > 0.05). BMI, WC, SBP, DBP, FBG, TG, uric acid levels, Wbc, Neu, Lym counts were significantly higher in the MetS group (all *p* < 0.001). HDL-C levels were significantly lower in MetS (*p* < 0.001), whereas LDL-C levels were significantly lower in the MetS group (*p* < 0.001). ALT levels were significantly higher in MetS (*p* < 0.001), while AST did not differ between groups (*p* = 0.346).

Comparisons of carotid stiffness and strain parameters are summarized in Table 2. In the longitudinal plane, patients with MetS demonstrated significantly higher stiffness indices, including β-SI, EM, and PWV, compared with controls (*p* = 0.013; *p* < 0.001; *p* < 0.001, respectively). Correspondingly, compliance parameters (AC and AD) were significantly reduced in the MetS group (*p* = 0.008 and *p* < 0.001, respectively). Among strain parameters, radial strain percentage was modestly lower in MetS (*p* = 0.043), whereas DP and SR did not differ significantly.

**Fig. 2:**
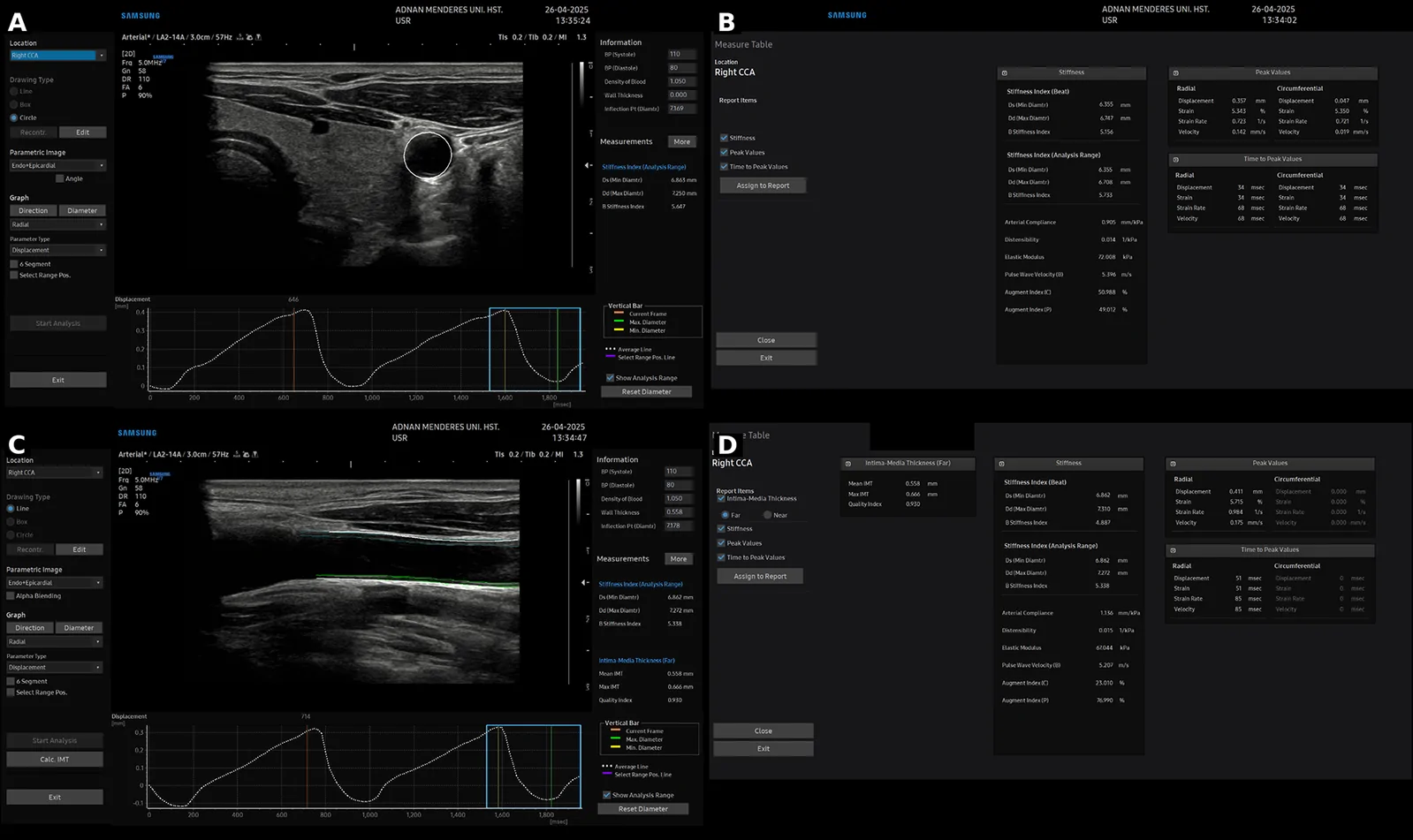
Representative STCS-US examination in a control participant. (**A**) Transverse-plane carotid ultrasound image with speckle-tracking analysis. (**B**) Transverse-plane stiffness and strain measurements generated by the Arterial Analysis software program. (**C**) Longitudinal-plane carotid ultrasound image with speckle-tracking analysis. (**D**) Longitudinal-plane stiffness and strain measurements generated by the Arterial Analysis software program

**Fig. 3:**
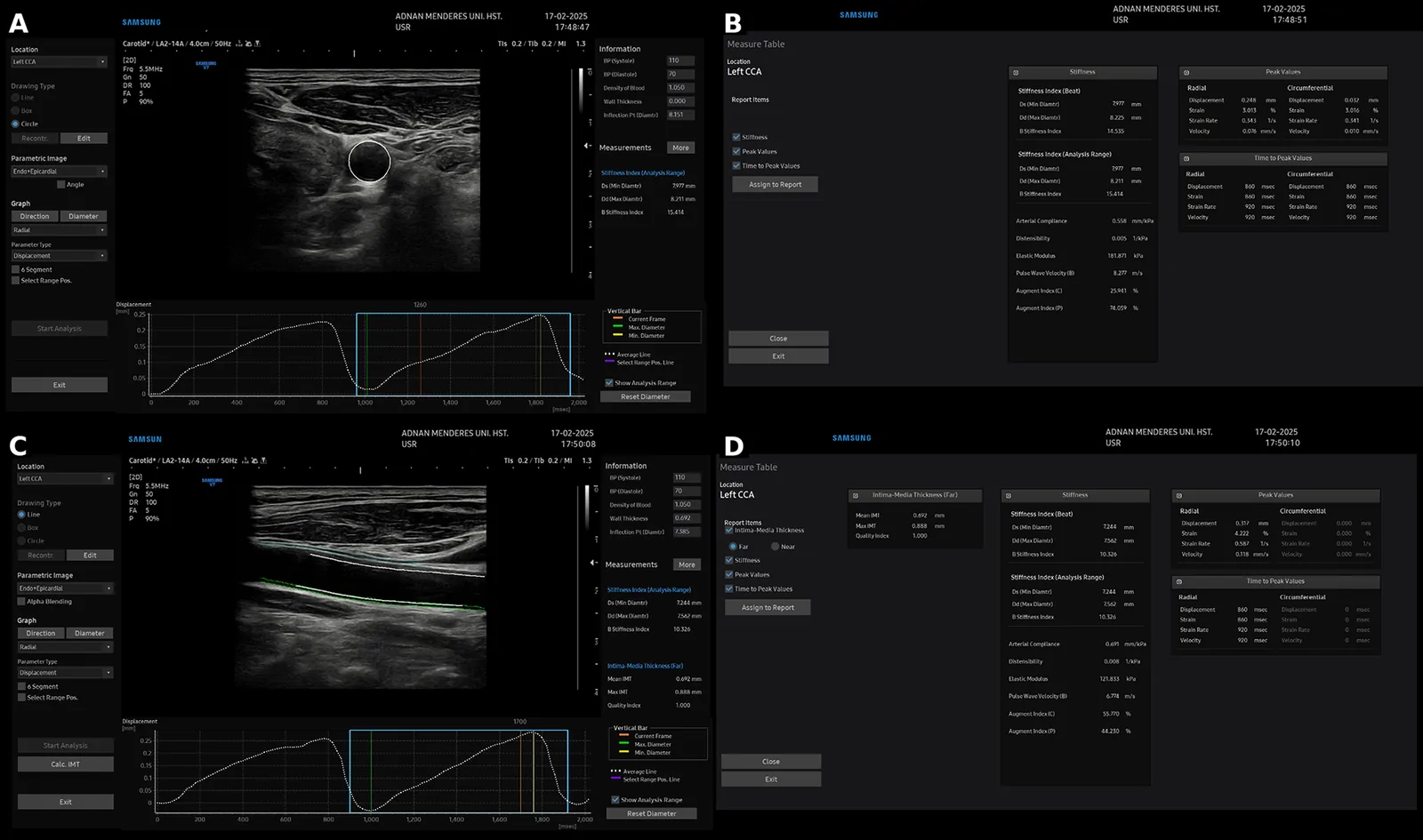
Representative STCS-US examination in a participant with metabolic syndrome. (**A**) Transverse-plane carotid ultrasound image with speckle-tracking analysis. (**B**) Transverse-plane stiffness and strain measurements generated by the Arterial Analysis software program. (**C**) Longitudinal-plane carotid ultrasound image with speckle-tracking analysis. (**D**) Longitudinal-plane stiffness and strain measurements generated by the Arterial Analysis software program

Alterations were more pronounced in the transverse plane. All stiffness parameters (β-SI, EM, and PWV) were significantly higher in the MetS group (all *p* < 0.001), accompanied by reduced AC and AD (*p* = 0.018 and *p* < 0.001, respectively). In addition, both radial and circumferential strain percentages were significantly decreased in MetS (both *p* < 0.001). Radial DP showed a modest reduction (*p* = 0.025), while SR parameters in both directions remained comparable between groups (all *p* > 0.05). Representative STCS-US examinations obtained from a control participant and a patient with MetS are shown in Figs. 2 and 3. The intra-observer reproducibility analysis demonstrated good agreement (ICC = 0.800, CV = 7.8%). As shown in Table 3, the number of MetS diagnostic criteria was significantly associated with multiple vascular parameters. Positive correlations were observed with stiffness indices, including β-SI, EM, and PWV, with stronger associations noted in the transverse plane (all *p* < 0.05). EM and PWV showed the strongest positive correlations, particularly in the transverse plane (all *p* < 0.001). In contrast, compliance indices (AC and AD) were inversely correlated with increasing MetS components (all *p* < 0.05).

Similarly, higher MetS burden was associated with reduced strain percentages. Both radial and circumferential strain demonstrated significant inverse correlations with the number of MetS criteria (both *p* < 0.001). No significant associations were observed for DP or SR parameters.

To further evaluate the relative contribution of clinical and structural variables to STCS-US-derived vascular parameters, CART analysis was performed. CART analysis was applied to all stiffness and strain parameters, and the results of interpretable CART models are presented in Table 4. MetS status was the most influential determinant of transverse-plane β-SI and EM, whereas BMI showed the highest relative importance for transverse-plane PWV. Age was the most influential determinant of both transverse-plane radial and circumferential strain. Importantly, MetS status also retained high relative importance in the PWV, radial strain, and circumferential strain models. CIMT, treatment-related variables, and smoking showed varying degrees of relative importance across the models, while plaque presence and sex generally had low importance scores. The corresponding decision tree diagrams illustrating the hierarchical splitting structure of each model are provided in Supplementary Figure S1.

## Discussion

In this study, MetS was associated with multidimensional impairment in carotid arterial mechanics, characterized by increased stiffness and reduced deformation capacity assessed by STCS-US. Importantly, these alterations were not confined to a single mechanical domain; both stiffness indices and strain parameters were associated with an increasing number of MetS components. This association aligns with prior evidence demonstrating that arterial stiffness increases with clustering of metabolic risk factors [12–15] and supports the biological plausibility of a cumulative metabolic burden on arterial wall function. Taken together, these findings suggest that STCS-US-derived metrics reflect not only the presence of MetS but also the degree of metabolic burden imposed on the arterial wall.

Previous studies have consistently demonstrated an association between MetS and arterial stiffness, predominantly using carotid–femoral pulse wave velocity (PWV) as a surrogate marker of vascular injury [5, 12, 13]. However, PWV primarily reflects global arterial rigidity and does not capture dynamic wall deformation during the cardiac cycle [6]. In contrast, STCS-US enables simultaneous evaluation of stiffness parameters (β-SI, EM, PWV, AC, AD) and strain parameters (radial and circumferential strain), providing complementary mechanical information within a single examination. While PWV reflects global arterial rigidity, strain parameters provide insight into dynamic wall deformation. The combined impairment observed in our study suggests that STCS-US may capture complementary dimensions of vascular dysfunction rather than merely replicating PWV findings. Our findings suggest that MetS affects not only arterial rigidity but also the capacity of the vessel wall to deform, indicating a broader disturbance in vascular mechanics.

The observation that strain parameters were significantly reduced particularly in the transverse plane and inversely correlated with the number of MetS components may indicate that deformation abnormalities parallel or potentially precede overt structural remodeling. Previous STCS-US studies have suggested that strain-based vascular parameters may detect subclinical vascular abnormalities before overt structural changes become apparent and may provide information beyond conventional stiffness measures [7, 9]. In addition, strain-based parameters have been reported to carry prognostic information beyond conventional stiffness measures [8]. Taken together, the combined impairment in stiffness and strain domains may suggest that metabolic clustering exerts an early functional impact on the arterial wall.

From a clinical perspective, MetS represents a heterogeneous entity with variable cardiovascular risk [2, 3]. While traditional risk assessment tools incorporate demographic and biochemical parameters, they do not directly evaluate vascular function. A technique capable of characterizing multiple aspects of arterial mechanics during a single non-invasive examination may offer a more comprehensive vascular phenotyping approach. The observed associations between stiffness and strain parameters and increasing numbers of MetS components suggest that STCS-US may capture gradations of vascular impairment beyond a simple binary classification. Although our study was not designed to evaluate prognostic performance, the multidimensional abnormalities detected by STCS-US raise the hypothesis that integrated mechanical assessment could contribute to improved vascular characterization in selected high-risk individuals.

The mechanisms underlying these findings are consistent with established pathophysiological pathways. Insulin resistance, oxidative stress, endothelial dysfunction, and chronic low-grade inflammation promote extracellular matrix remodeling and reduce arterial elasticity [16–18]. Hypertension and dyslipidemia further accelerate vascular remodeling and collagen deposition [16]. In our cohort, elevated triglycerides and reduced HDL-C were consistent with prior studies demonstrating associations between atherogenic lipid profiles and arterial stiffness [19, 20], reinforcing the concept that atherogenic dyslipidemia may better reflect vascular risk in MetS than LDL-C levels alone. Notably, LDL-C levels were lower in the MetS group, which we attribute primarily to the higher prevalence of lipid-lowering therapy in this population. Higher serum uric acid levels, previously linked to increased arterial stiffness [21–23], and elevated leukocyte indices, associated with vascular dysfunction [24, 25], further support a systemic metabolic–inflammatory milieu contributing to impaired arterial mechanics. Additionally, the higher ALT levels observed in MetS participants may reflect underlying metabolic liver dysfunction, which has been independently associated with increased arterial stiffness [26].

STCS-US has been shown to be reproducible and feasible in different clinical contexts. Prior studies demonstrated its utility in detecting early vascular dysfunction in type 1 diabetes [7] and its prognostic relevance in metabolic populations [8]. Furthermore, speckle-tracking techniques have been highlighted as valuable tools for integrating stiffness and strain analysis within vascular imaging [27, 28]. In this context, STCS-US should not be viewed solely as a technical refinement but rather as a method enabling multidimensional assessment of arterial wall mechanics within a single examination.

Importantly, this study was not designed to determine whether STCS-US is superior to conventional PWV-based assessment or whether it provides incremental diagnostic or prognostic value beyond established stiffness measures. Direct head-to-head performance comparisons, reclassification analyses, and outcome-based validation were beyond the scope of the present investigation. Therefore, STCS-US should be interpreted as a complementary tool that captures additional dimensions of arterial wall mechanics rather than as a replacement for conventional vascular stiffness assessment.

Several limitations warrant consideration. The retrospective design precludes causal inference, and the study was not powered to assess clinical outcomes. Incremental diagnostic or prognostic performance beyond conventional stiffness indices was not evaluated. Differences in medication exposure between groups may introduce residual confounding. In particular, antihypertensive, lipid-lowering, and antidiabetic therapies may influence vascular strain and stiffness parameters and may have attenuated the magnitude of the observed differences between groups. Although CIMT and plaque presence were included in the analysis, the relationship between structural vascular changes and STCS-US-derived functional parameters was not specifically investigated. Intra-observer reproducibility analysis of offline measurements demonstrated good agreement. However, inter-observer measurement reproducibility was not evaluated. In addition, because STCS-US measurements were retrospectively derived from archived cine-loop images, the reproducibility of image acquisition by the same or different operators could not be assessed. Future prospective studies using standardized acquisition protocols should evaluate both measurement reproducibility and image-acquisition reproducibility. Finally, the single-center design and modest sample size restrict generalizability. Accordingly, these findings should be considered hypothesis-generating and require confirmation in prospective, multicenter cohorts.

## Conclusions

Metabolic syndrome is associated with coordinated impairment in carotid arterial stiffness and dynamic deformation assessed by STCS-US. Alterations were more pronounced in the transverse plane, and both stiffness and strain parameters were associated with an increasing number of MetS components. These findings indicate that STCS-US captures multidimensional aspects of vascular dysfunction beyond conventional stiffness metrics alone. Although prospective outcome-based studies are required, STCS-US may serve as a complementary tool for functional vascular assessment in individuals with metabolic syndrome.

**Table 1:** Comparison of demographic characteristics and laboratory parameters between patients with metabolic syndrome (MetS) and the control group

Parameter	MetS (*n* = 50)	Control (*n* = 50)	*p*
Age (years)	52.1 ± 8.8	51.1 ± 9.6	0.588
Sex, n (%)	26 female (52), 24 male (48)	31 female (62), 19 male (38)	0.313
History of smoking, n (%)	28 (56)	25 (50)	0.548
Hypertension, n (%)	33 (66)	7 (14)	**< 0.001**
Diabetes mellitus, n (%)	34 (68)	2 (4)	**< 0.001**
Dyslipidemia, n (%)	26 (52)	0 (0)	**< 0.001**
Antihypertensive use, n (%)	32 (64)	7 (14)	**< 0.001**
Antidiabetic use, n (%)	34 (68)	2 (4)	**< 0.001**
Antihyperlipidemic use, n (%)	25 (50)	0 (0)	**< 0.001**
BMI (kg/m²)	32.6 ± 5.1	25.9 ± 4.2	**< 0.001**
WC (cm)	110.0 (100.0-120.0)	87.0 (82.0-98.5)	**< 0.001**
SBP (mmHg)	130.0 (120.0-140.0)	120.0 (110.0-120.0)	**< 0.001**
DBP (mmHg)	80.0 (73.7–90.0)	70.0 (70.0–75.0)	**< 0.001**
Hemoglobin (g/dL)	13.7 (12.7–15.3)	13.6 (12.7–14.6)	0.515
Platelet (×10⁹/L)	268.8 ± 78.3	258.1 ± 67.7	0.468
Leukocyte (×10³/mm³)	8.4 ± 2.5	6.8 ± 1.9	**< 0.001**
Neutrophil (×10³/mm³)	4.9 (4-5.9)	4.2 (2.8–4.8)	**< 0.001**
Lymphocyte (×10³/mm³)	2.7 (2.3–3.4)	2.1 (1.7–2.5)	**< 0.001**
FBG (mg/dL)	113.0 (92.8–158.0)	88.0 (84.0-93.3)	**< 0.001**
BUN (mg/dL)	12.2 (10.8–14.7)	11.2 (7.5–14.0)	0.051
Creatinine (mg/dL)	0.8 (0.7–0.9)	0.7 (0.6–0.9)	0.173
eGFR (mL/min/1.73 m²)	98.2 (91.2-106.4)	99.8 (89.2-109.8)	0.374
Total cholesterol (mg/dL)	208.7 ± 34.6	216.6 ± 44.5	0.293
LDL-C (mg/dL)	114.1 ± 35.0	142.8 ± 39.0	**< 0.001**
HDL-C (mg/dL)	41.5 (35.0-48.3)	52.1 (44.0–59.0)	**< 0.001**
Triglycerides (mg/dL)	245.5 (137.3–322.0)	90.5 (77.0-108.0)	**< 0.001**
ALT (U/L)	21.5 (16.0-31.5)	15.0 (12.0-20.3)	**< 0.001**
AST (U/L)	20.0 (15.8–26.3)	19.0 (16.0–22.0)	0.346
Uric acid (mg/dL)	5.9 ± 1.3	4.5 ± 1.6	**< 0.001**

BMI: body mass index, SBP: systolic blood pressure, DBP: diastolic blood pressure, FBG: fasting blood glucose, BUN: blood urea nitrogen, eGFR: estimated glomerular filtration rate, LDL−C: low−density lipoprotein cholesterol, HDL−C: high−density lipoprotein cholesterol, ALT: alanine aminotransferase, AST: aspartate aminotransferase

**Table 2:** Comparison of radiological parameters between patients with metabolic syndrome (MetS) and the control group

Parameter	MetS (*n* = 50)	Control (*n* = 50)	*p*
Plaque presence, n (%)	9 (18)	3 (6)	0.124
CIMT (mm)	0.729 (0.653–0.807)	0.604 (0.560–0.707)	**< 0.001**
**Longitudinal Plane**			
**Stiffness parameters**			
β-SI	8.652 (5.995–9.912)	6.633 (5.416–8.422)	**0.013**
AC(mm/kPa)	0.531 (0.419–0.736)	0.677 (0.538–0.934)	**0.008**
AD(kPa⁻¹)	0.008 (0.007–0.011)	0.011 (0.010–0.015)	**< 0.001**
EM(kPa)	136.776 (102.504-165.212)	88.831 (68.194–109.670)	**< 0.001**
PWV(m/s)	6.770 ± 1.134	5.789 ± 0.995	**< 0.001**
**Strain parameters (Radial)**			
DP(mm)	0.372 ± 0.094	0.406 ± 0.115	0.110
Strain(%)	5.569 (4.817–6.286)	6.347 (4.787–7.940)	**0.043**
SR(s⁻¹)	0.856 (0.648–0.994)	0.797 (0.682–1.014)	0.997
**Transverse Plane**			
**Stiffness parameters**			
β-SI	10.340 ± 2.937	7.502 ± 1.756	**< 0.001**
AC(mm/kPa)	0.620 (0.506–0.748)	0.699(0.589–0.925)	**0.018**
AD(kPa⁻¹)	0.007 (0.006–0.008)	0.010 (0.009–0.013)	**< 0.001**
EM(kPa)	159.373 ± 44.219	92.134 ± 28.864	**< 0.001**
PWV(m/s)	7.391 ± 1.086	6.096 ± 0.914	**< 0.001**
**Strain parameters (Radial)**			
DP(mm)	0.331 (0.293–0.396)	0.388 (0.319–0.460)	**0.025**
Strain(%)	4.296 (3.916–5.504)	5.744 (4.792–6.857)	**< 0.001**
SR(s⁻¹)	0.615 (0.505–0.789)	0.710 (0.575–0.812)	0.178
**Strain parameters (Circumferential)**			
DP(mm)	0.043 (0.039–0.052)	0.050 (0.041–0.060)	0.058
Strain(%)	4.298 (3.923–5.412)	5.719 (4.767–6.804)	**< 0.001**
SR(s⁻¹)	0.620 (0.501–0.793)	0.703 (0.558-0.800)	0.224

CIMT: carotid intima−media thickness, β−SI: beta stiffness index, AC: arterial compliance, AD: arterial distensibility, EM: elastic modulus, PWV: pulse wave velocity, DP: displacement, SR: strain rate

**Table 3:** Correlation between the number of metabolic syndrome (MetS) criteria and arterial stiffness and strain parameters

Parameter	MetS criteria count	
	*r*	*p*
**Longitudinal Plane**		
**Stiffness parameters**		
β-SI	0.220	**0.028**
AC(mm/kPa)	-0.219	**0.029**
AD(kPa⁻¹)	-0.371	**< 0.001**
EM(kPa)	0.419	**< 0.001**
PWV(m/s)	0.383	**< 0.001**
**Strain parameters (Radial)**		
DP(mm)	-0.089	0.379
Strain(%)	-0.166	0.100
SR(s⁻¹)	0.028	0.784
**Transverse Plane**		
**Stiffness parameters**		
β-SI	0.510	**< 0.001**
AC(mm/kPa)	-0.227	**0.023**
AD(kPa⁻¹)	-0.603	**< 0.001**
EM(kPa)	0.684	**< 0.001**
PWV(m/s)	0.585	**< 0.001**
**Strain parameters (Radial)**		
DP(mm)	-0.174	0.084
Strain(%)	-0.368	**< 0.001**
SR(s⁻¹)	-0.136	0.177
**Strain parameters (Circumferential)**		
DP(mm)	-0.141	0.162
Strain(%)	-0.371	**< 0.001**
SR(s⁻¹)	-0.104	0.304

β−SI: beta stiffness index, AC: arterial compliance, AD: arterial distensibility, EM: elastic modulus, PWV: pulse wave velocity, DP: displacement, SR: strain rate

**Table 4:** Normalized variable importance (%) of candidate determinants in CART models for selected STCS-US-derived vascular parameters

Variable	β-SI (Transverse)	EM (Transverse)	PWV (Transverse)	Radial Strain (Transverse)	Circumferential Strain (Transverse)
MetS status	100	100	92.9	87.4	93.1
Age	69.5	43.0	57.5	100	100
Sex	1.0	7.0	6.5	16.5	20.9
BMI	75.6	69.2	100	67.8	68.0
Smoking	2.5	1.3	5.2	42.8	46.8
CIMT	56.4	45.4	64.9	67.9	69.9
Plaque presence	3.3	2.1	1.1	6.9	8.3
Antidiabetic treatment	54.7	47.5	46.2	67.7	67.0
Antihypertensive treatment	40.2	49.5	43.8	20.1	17.6
Antihyperlipidemic treatment	11.3	23.6	13.7	18.5	20.4
MAE	1.57	24.08	0.64	1.01	0.99
MAPE (%)	18.81	25.44	10.29	19.99	19.57

Values represent normalized importance scores, with the candidate determinant having the highest relative importance in each model assigned a value of 100%. β−SI: beta stiffness index, EM: elastic modulus, PWV: pulse wave velocity, BMI: body mass index; CIMT: carotid intima−media thickness; MetS: metabolic syndrome, MAE: mean absolute error

## Supplementary Information

Below is the link to the electronic supplementary material.

Supplementary Material 1

## Data Availability

The datasets generated and/or analysed during the current study are not publicly available due to institutional regulations and the protection of patient privacy but are available from the corresponding author on reasonable request, subject to approval by the local ethics committee.
