# Supplementary material for: Speckle-tracking carotid strain ultrasonography for assessment of vascular dysfunction in metabolic syndrome: a cross-sectional study
Source: BMC Med Imaging. 2026 Sep 11;26:445. doi: 10.1186/s12880-026-02686-5 (PMC13563693; doi:10.1186/s12880-026-02686-5)
Supplement: Supplementary file 1 [file 12880_2026_2686_MOESM1_ESM.docx]

**Supplementary Figure S1. CART models for selected STCS-US-derived vascular parameters. (A) β-stiffness index (β-SI, transverse plane); (B) Elastic modulus (EM, transverse plane); (C) Pulse wave velocity (PWV, transverse plane); (D) Radial strain (transverse plane); (E) Circumferential strain (transverse plane).**

| A |  |
| --- | --- |
| B |  |
| C |  |
| D |  |
| E |  |
